# Metataxonomic Analysis of Milk Samples From SARS-CoV-2-Positive and SARS-CoV-2-Negative Women

**DOI:** 10.3389/fnut.2022.853576

**Published:** 2022-03-18

**Authors:** Natalia Gómez-Torres, Laura Sánchez-García, Irma Castro, Rebeca Arroyo, Fernando Cabañas, Raquel González-Sánchez, Manuela López-Azorín, M. Teresa Moral-Pumarega, Diana Escuder-Vieco, Esther Cabañes-Alonso, Juan Miguel Rodríguez, Claudio Alba, Adelina Pellicer

**Affiliations:** ^1^Department of Nutrition and Food Science, Complutense University of Madrid, Madrid, Spain; ^2^Department of Neonatology, Biomedical Research Foundation-IDIPAZ, La Paz University Hospital, Madrid, Spain; ^3^Department of Neonatology, Quirónsalud Madrid University Hospital and Quirónsalud San José Hospital, Biomedical Research Foundation-IDIPAZ, La Paz University Hospital, Madrid, Spain; ^4^Department of Neonatology, Quirónsalud Madrid University Hospital and Quiroónsalud San José Hospital, Madrid, Spain; ^5^Department of Neonatology, 12 de Octubre University Hospital, Madrid, Spain; ^6^Department of Neonatology, Regional Human Milk Bank, 12 de Octubre University Hospital, Madrid, Spain

**Keywords:** SARS-CoV-2, human milk, microbiota, metataxonomic analysis, infant gut colonization

## Abstract

**Objective:**

To assess the impact of SARS-CoV-2 viral infection on the metataxonomic profile and its evolution during the first month of lactation.

**Methods:**

Milk samples from 37 women with full-term pregnancies and mild SARS-CoV-2 infection and from 63 controls, collected in the first and fifth postpartum weeks, have been analyzed. SARS-CoV-2 RNA was assessed by reverse transcription polymerase chain reaction (RT-PCR) both in cases and controls. After DNA extraction, the V3-V4 hypervariable region of the gene 16S rRNA was amplified and sequenced using the MiSeq system of Illumina. Data were submitted for statistical and bioinformatics analyses after quality control.

**Results:**

All the 1st week and 5th week postpartum milk samples were negative for SARS-CoV-2 RNA. Alpha diversity showed no differences between milk samples from the study and control group, and this condition was maintained along the observation time. Analysis of the beta-diversity also indicated that the study and control groups did not show distinct bacterial profiles. *Staphyloccus* and *Streptococcus* were the most abundant genera and the only ones that were detected in all the milk samples provided. Disease state (symptomatic or asymptomatic infection) did not affect the metataxonomic profile in breast milk.

**Conclusion:**

These results support that in the non-severe SARS-CoV-2 pregnant woman infection the structure of the bacterial population is preserved and does not negatively impact on the human milk microbiota.

## Introduction

The possibility of mother-to-infant transmission of SARS-CoV-2 during pregnancy and breastfeeding was a matter of concern at the start of the COVID-19 pandemic. The first question to address about the impact of SARS-CoV-2 infection on breastfeeding was if the virus was present or not in human colostrum or mature milk samples, with a few studies finding positive samples (1) but most of them showing negative results (2–8). Second, the research focused on testing whether these biological fluids carry anti-SARS-CoV-2 antibodies with the property of protecting nursing babies from infection (4, 9, 10).

SARS-CoV-2 initially interacts with the mucosal surface of the upper respiratory tract (11, 12) and with that of the gastrointestinal tract (13), which are associated with a highly complex microbiota. SARS-CoV-2 infection pathophysiology is related to the angiotensin-converting enzyme receptor 2 (ACE2), whose activity is influenced and, in turn, influences the microbiota (14). These facts suggest an implication of the host microbiota in the modulation of SARS-CoV-2 infection (15). Some studies have already found that the presence of key immunomodulatory species in the gut microbiota, such as bifidobacteria, *Faecalibacterium prausnitzii*, or *Eubacterium rectale*, is reduced in COVID-19 patients (16, 17), and the magnitude of this change is closely associated to disease severity (18, 19). It has been reported that the gut dysbiosis usually persists beyond disease state resolution and may contribute to the long-lasting COVID-19 sequelae (18, 19). The modulatory role of the microbiota has already been observed with other respiratory viruses, including other coronaviruses and the respiratory syncytial virus (20–22).

Breast milk contains a site-specific microbiota (23), which is dominated by species of the genera *Staphylococcus*, *Streptococcus*, *Corynebacterium*, and *Cutibacterium*, although lactic acid bacteria and bifidobacteria can also be isolated. The microbiome of human milk seems to be a dynamic and complex ecosystem which is not randomly assembled but forms well-organized bacterial consortia and networks (24). This microbiome and its evolution along the lactation period are relevant from both the scientific and biomedical points of view since human milk-associated microbes may determine, at least partly, the pattern of gastrointestinal colonization during early life (25, 26). Breast milk bacteria not only drive infant gut colonization but, also, they may play other important biological functions, such as protecting against pathogens, influencing the development of the mucosal immune system, and helping digestion and nutrients’ absorption (27, 28). However, there are many maternal, neonatal and environmental factors that can affect the composition of the milk microbiome over time (29–31). Since colostrum and mature milk microbes are among the first colonizers of the human gut, any factor disturbing the microbiome profile of these biological fluids may potentially impact infant gut colonization and future health. Interestingly, DNA from some gut-associated strict anaerobes which presence is reduced in COVID-19 patients (*Faecalibacterium* or *Eubacterium*, among others) has also been detected in human milk using culture-independent techniques (32–36).

On April 2020, a multidisciplinary group designed a research plan to foster the knowledge on breastfeeding and SARS-CoV-2 infection, with the aims of providing data on its safety, i.e., exclusion of human milk as a source of virus spread, and its efficacy in the transfer of protection factors against SARS-CoV-2 infection. The purpose of this work is to report on the impact of SARS-CoV-2 viral infection on the breast milk metataxonomic profile and its evolution during the first month of lactation.

## Materials and Methods

A multicenter, prospective case and control study was conducted in four maternity hospitals of Madrid, which gather a total number of deliveries per year above 13.000. Since March 2020, all pregnant women underwent routine nasopharyngeal SARS-CoV-2 Reverse Transcription Polymerase Chain Reaction (RT-PCR) test as screening upon admission prior delivery. Recruitment was completed between April and July 2020. The study was approved by the referral Clinical Research Ethics Committee (La Paz University Hospital). Informed consent was obtained from mothers before enrollment. Every mother-infant’ information was treated anonymously.

### Study Procedures

Term pregnant women with confirmed SARS-CoV-2 infection at the time of delivery were approached, providing they were in good clinical condition and had a decision to breastfeed their healthy babies (study group; *n* = 45). Consecutive sample (1case:2controls) of SARS-CoV-2 negative pregnant women, who were in identical conditions, was obtained (control group; *n* = 96). Prospective clinical data of participant mothers and their infants were recorded.

Women were instructed on how to extract and store milk to avoid contaminations. Milk samples were immediately frozen at −20°C and shipped on dry ice (−78.5°C) to the Department of Nutrition and Food Science, Complutense University of Madrid, for further processing. Breast milk samples collected during the first- and fifth-week postpartum were used for analyses.

### Reverse Transcription Polymerase Chain Reaction Assays

RNA extraction from the milk samples (200 μL) was carried out using the KINGFISHER FLEX 96 extraction robot (Thermo Fisher Scientific), the MagMax_Core_Flex extraction program and the MagMAX Viral/Pathogen II Nucleic Acid Isolation kit (Applied Biosystems, Thermo Fisher Scientific). For the detection of SARS-CoV-2, the TaqPath COVID-19 CE-IVD RT-PCR kit (Applied Biosystems, Thermo Fisher Scientific) was used in a 384-well format with the QuantStudio 7 Flex System equipment (Applied Biosystems). In this kit, probes anneal to three target sequences (ORF1ab, N Protein and S Protein genes) that are specific to SARS-CoV-2. All lab kits were used following the manufacturer’s instructions.

### DNA Extraction, Metataxonomic and Bioinformatic Analysis

DNA extraction from the milk samples (1 mL) was performed as described by Lackey et al. (37). The V3-V4 hypervariable region of the gene 16S rRNA was amplified and sequenced using the MiSeq system of Illumina at the facilities of Parque Científico de Madrid (Tres Cantos, Spain) with the Illumina MiSeq 2 × 300 bp pair-end protocol (Illumina Inc., San Diego, CA, United States) (38).

Raw sequences data were demultiplexed and quality filtered with the Illumina MiSeq Reporter analysis software. Bioinformatic analysis was performed with QIIME 2 2019.1 (39) pipelines. The denoising step was performed with DADA2 (40). The forward reads were truncated at position 295 and trimming the first 12 nucleotides, while the reverse sequences were truncated at the 275 nucleotides and trimming the first 9 nucleotides, in order to discard nucleotides which median quality were Q20 or below. Also, all amplicon sequence variants (ASVs) were classified in a feature count table. Taxonomy was assigned to each ASVs with the q2-feature-classifier (41) classify-sklearn, naïve Bayes taxonomy classifier, using the SILVA 138 reference database (42). Subsequent bioinformatic analysis was conducted using R version 3.5.1 (R Core Team, 2013).^1^ The decontam package (43) was used in order to identify, visualize and remove contaminating DNA.

ASVs, genera and phyla sequence count tables per sample were generated, and bacterial taxa abundances were normalized to the total number of sequences in each sample (relative abundance). Alpha diversity was assessed using the Shannon and Simpson diversity indexes. For the beta diversity studies, a quantitative (relative abundance) and a qualitative (presence/absence) analysis, for the bacterial profiles, were performed with the Bray-Curtis dissimilarity and binary Jaccard indices, respectively. Principal Coordinates Analysis (PCoA) was performed in order to plot patterns of bacterial profiles through the Bray-Curtis and binary Jaccard distance matrices.

A set of “core” genera were characterized for each group. To be included in the core taxa, a genus must have been present in ≥ 90% of the samples with a representability ≥ 0.1% of the relative abundance of each sample, for one or two groups. The 5 most-abundant phyla from all the milk samples were selected as most abundance phyla and the rest were included in the “minor_phyla” group. The 19 most-abundant genera from all the milk samples were selected as most abundance genera and the rest were included in the “minor_genera” group.

### Statistical Analysis

Demographic data were expressed as mean and standard deviation (SD) and differences between study and control groups were assessed using the Fisher exact (or Chi-squared) and the *t*-test for categorical and quantitative variables, respectively.

Quantitative data on microbiota were expressed as median and interquartile range (IQR). Differences between study and control groups were assessed using the Wilconxon rank test. PCoA and the PERMANOVA analysis with 999 permutations were performed to examine for similarities in the bacterial profile between groups. Statistical analysis and plotting were performed in the R environment using R version 3.5.1 (R Core Team, 2013) (see text footnote 1) with library ggplot2 (44). Differences were considered statistically significant at *p* < 0.05.

## Results

**FIGURE 1:**
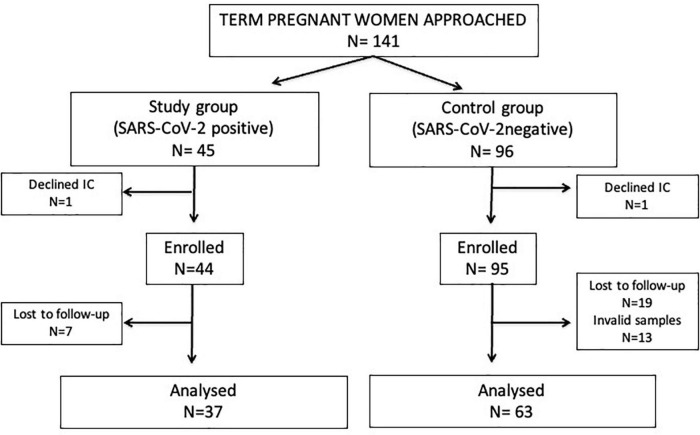
Flow diagram of the study participants. IC, informed consent. Invalid samples: non-availability, inadequate patient identification or inadequate handling or storage.

Forty-five term pregnant women with confirmed non-severe SARS-CoV-2 infection and uncomplicated delivery (study group), and 96 SARS-CoV-2 negative women in identical conditions (control group) were approached. Of them, 37 study group and 63 control group women were included in the final analyses. All women had normal weight (body mass index: 19–24) with only one exception (one woman of the study group was overweighed). Details on participants’ chart flow and reasons for exclusion are described on Figure 1.

No differences between the study and control group were found regarding maternal age (33.9 ± 5.4 years vs. 34.5 ± 4.4 years, *p* = 0.56), prevalence of previous maternal health problems [6 (16%) vs. 10 (16%), *p* = 0.99; including the case of overweight in the study group], rates of vaginal delivery [26 (70%) vs. 48 (76%), *p* = 0.85], gestational age (39.1 ± 1.7 weeks vs. 38.9 ± 1.9 weeks, *p* = 0.64) and birth weight (3,187 ± 543 g vs. 3,179 ± 536 g, *p* = 0.94) of infants.

Mild SARS-CoV-2 infection related symptoms were present in 21 (56.8%) of study group women, including fever (48%), anosmia (48%), cough (43%), ageusia (14%), odynophagia (10%), myalgia (10%), diarrhea (10%), or headache (5%); and 19 out of the 21 symptomatic women received medication (anticoagulation, antibiotics, hydroxychloroquine, oxygen therapy) around labor. None of the infants of mothers in the study or the control group presented clinical signs associated to SARS-COV-2 infection within the first month of life. By hospital protocol, nasopharyngeal RT-PCR was performed in neonates of positive SARS-COV-2 mothers, resulting negative in all cases.

All the 1st week and 5th week postpartum samples collected and processed were negative for SARS-CoV-2 RNA (study group: 36 samples at 1st week and 37 samples at 5th week postpartum; control group: 63 samples at 1st week and 63 samples at 5th week postpartum), that were submitted to metataxonomic analysis. Among them, 197 milk samples (study group: 36 at 1st week, 37 at 5th week; control group: 62 at 1st and 5th week) showed a distinct single electrophoretic band after the first PCR round; the size of these amplicons matched the exact size of the V3-V4 hypervariable region and, therefore, they were selected for the second PCR round and sequencing. The 16S rRNA gene sequencing yielded 12,471,894 high quality filtered sequences, ranging from 21,862 to 183,796 reads per sample [median sequence per sample 60,067 (51,499–70,938)] with a total of 5,003 ASVs.

Alpha diversity showed no differences between milk samples from the study and control group, and this condition was maintained throughout the observation period (Table 1).

**TABLE 1:** Bacterial diversity in breast milk samples.

	Study group			Control group			*P*-value^†^				
	Group diversity median (IQR)	1st week median (IQR)	5th week median (IQR)	Group diversity median (IQR)	1st week median (IQR)	5th week median (IQR)	(a)	(b)	(c)	(d)	(e)
Shannon index	2.3 (1.84–2.66)	2.16 (1.53–2.38)	2.27 (1.97–2.67)	2.2 (1.82–2.52)	2.19 (1.78–2.63)	2.31 (1.93–2.72)	0.210	0.98	0.99	1	1
Simpson index	0.83 (0.71–0.87)	0.80 (0.66–0.84)	0.82 (0.77–0.88)	0.8 (0.73–0.86)	0.78 (0.70–0.86)	0.84 (0.74–0.87)	0.610	0.53	0.87	1	1

*^†^Pairwise comparisons using Wilcoxon rank sum test with Bonferroni correction: (a) study vs. control group; within group comparison 1st vs. 5th week:(b) study group and (c) control group; between group comparison: (d) 1st week and (e) 5th week.*

**FIGURE 2:**
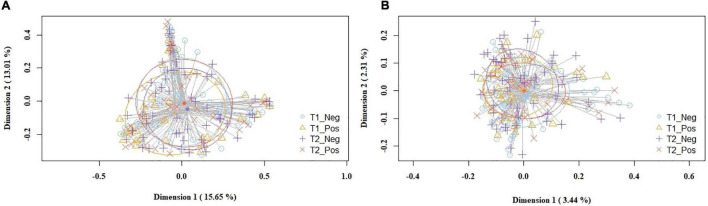
PCoA plots of bacterial profiles (at the ASVs level) of milk samples according to SARS-CoV-2 status (study and control group) and study time point, based on the Bray-Curtis similarity analysis (relative abundance) **(A)**, and Jaccard’s coefficient for binary data (presence/absence) **(B)**. Where blue circles (T1_Neg) refer to 1st week samples from the control group, yellow triangles (T1_Pos) to 1st week samples from the study group, purple crosses (T2_Neg) to 5th week samples from the control group, and orange × s (T2_Pos) to 5th week samples from the study group. The value given on each axis label represents the percentage of the total variance explained by that axis.

The overall analysis of the beta diversity indicated that the profiles of bacterial genera of the study and control groups did not show distinct bacterial profiles. The PCoA plots of the Bray-Curtis distance matrix (relative abundance) yielded no differences between groups (*p* = 0.163), while the binary Jaccard distance matrix revealed small but statistically significant differences (*p* = 0.016) in the presence/absence of bacterial taxa between the study and the control groups (Figure 2).

The frequency of the most abundant bacterial phyla and genera detected in milk samples according to group and observation time is displayed on Table 2, indicating a high similarity in the relative abundance between groups, with a few exceptions; among them, the relative abundance of the genus *Gemella* was higher among the samples of the control group. *Staphylococcus* and *Streptococcus* were the most abundant genera and the only ones that were detected in all the milk samples processed. No differences regarding diversity or any taxonomic level were found in relation to disease expression in SARS-CoV-2-positive women (symptomatic vs. asymptomatic infected women).

**TABLE 2:** Frequency of the most abundant bacterial phyla and genera detected in milk samples according to group and study time point.

	Study group (1st week)	Study group (5th week)	Control group (1st week)	Control group (5th week)
Firmicutes*	36 (100%) 89.81 (75.74–95.87)	37 (100%) 79.24 (17.88–94.62)	62 (100%) 90.66 (74.14–96.57)	62 (100%) 73.91 (25.53–95.07)
*Streptococcus*	36 (100%) 22.52 (2.79–49.35)	37 (100%) 20.79 (0.97–56.47)	62 (100%) 37.91 (9.78–61.31)	62 (100%) 21.15 (2.82–49.58)
*Staphylococcus*	36 (100%) 39.09 (5.67–69.43)	37 (100%) 8.6 (2.76–19.05)	62 (100%) 20.85 (5.81–61.75)	62 (100%) 16.4 (2.63–37.61)
Gemella**	17 (47.22%) <0.01 (< 0.01–0.55)	24 (64.86%) 0.03 (< 0.01–0.60)	42 (67.74%) 0.22 (< 0.01–4.03)	50 (80.65%) 0.59 (0.02–3.23)
*Bacillus*	12 (33.33%) <0.01 (<0.01–0.04)	12 (32.43%) < 0.01 (< 0.01–0.04)	14 (22.58%) < 0.01 (< 0.01– < 0.01)	24 (38.71%) < 0.01 (< 0.01–0.05)
*Lactobacillus*	25 (69.44%) 0.05 (<0.01–0.28)	25 (67.57%) 0.04 (< 0.01–0.17)	35 (56.45%) 0.02 (< 0.01–0.24)	42 (67.74%) 0.04 (< 0.01–0.40)
*Enterococcus*	11 (30.56%) <0.01 (< 0.01–0.01)	12 (32.43%) < 0.01 (< 0.01–0.02)	11 (17.74%) < 0.01 (< 0.01– < 0.01)	15 (24.19%) < 0.01 (< 0.01– < 0.01)
*Lactococcus*	4 (11.11%) <0.01 (< 0.01– < 0.01)	7 (18.92%) < 0.01 (< 0.01– < 0.01)	6 (9.68%) < 0.01 (< 0.01– < 0.01)	8 (12.9%) < 0.01 (< 0.01– < 0.01)
Proteobacteria	36 (100%) 3.82 (0.99–13.52)	37 (100%) 10.80 (2.3–76.82)	62 (100%) 5.54 (2.1–17.45)	62 (100%) 16.64 (2.33–65.43)
*Acinetobacter*	32 (88.89%) 0.27 (0.06–1.03)	36 (97.3%) 1.30 (0.26–17.11)	53 (85.48%) 0.23 (0.07–1.39)	58 (93.55%) 0.63 (0.20–8.96)
*Pseudomonas*	30 (83.33%) 0.29 (0.03–1.19)	32 (86.49%) 0.76 (0.10–6.04)	54 (87.1%) 0.30 (0.06–2.39)	53 (85.48%) 0.78 (0.19–6.21)
*Stenotrophomonas*	13 (36.11%) <0.01 (<0.01–0.01)	22 (59.46%) 0.03 (<0.01–0.54)	22 (35.48%) <0.01 (< 0.01–0.05)	35 (56.45%) 0.03 (<0.01–0.66)
*Serratia*	12 (33.33%) <0.01 (<0.01–0.02)	7 (18.92%) <0.01 (<0.01–<0.01)	12 (19.35%) <0.01 (< 0.01–<0.01)	18 (29.03%) <0.01 (<0.01–0.02)
Allorhizobium*	9 (25%) <0.01 (<0.01–<0.01)	15 (40.54%) <0.01 (<0.01–0.1)	15 (24.19%) <0.01 (<0.01–<0.01)	30 (48.39%) <0.01 (<0.01–0.16)
*Sphingomonas*	18 (50%) <0.01 (<0.01–0.11)	16 (43.24%) <0.01 (<0.01–0.05)	20 (32.26%) <0.01 (<0.01–0.01)	30 (48.39%) <0.01 (<0.01–0.08)
*Enterobacter*	4 (11.11%) <0.01 (<0.01– < 0.01)	9 (24.32%) < 0.01 (< 0.01– < 0.01)	5 (8.06%) < 0.01 (< 0.01– < 0.01)	12 (19.35%) <0.01 (<0.01–<0.01)
Aquabacterium*	24 (66.67%) 0.02 (<0.01–0.20)	22 (59.46%) 0.01 (< 0.01–0.11)	42 (67.74%) 0.06 (< 0.01–0.39)	34 (54.84%) 0.01 (< 0.01–0.08)
*Sphingobium*	2 (5.56%) <0.01 (<0.01– < 0.01)	7 (18.92%) < 0.01 (< 0.01– < 0.01)	9 (14.52%) < 0.01 (< 0.01– < 0.01)	11 (17.74%) < 0.01 (< 0.01– < 0.01)
Actinobacteriota	34 (94.44%) 1.44 (0.41–3.66)	36 (97.3%) 0.40 (0.07–2.3)	58 (93.55%) 1.13 (0.23–4.04)	56 (90.32%) 0.69 (0.18–2.4)
*Rothia*	23 (63.89%) 0.17 (< 0.01–2.12)	24 (64.86%) 0.06 (< 0.01–0.87)	42 (67.74%) 0.30 (< 0.01–1.94)	35 (56.45%) 0.02 (< 0.01–0.34)
Bacteroidota	27 (75%) 0.05 (0.01–0.64)	28 (75.68%) 0.08 (0.01–1.57)	42 (67.74%) 0.08 (< 0.01–0.35)	49 (79.03%) 0.12 (0.01–0.33)
*Chryseobacterium*	15 (41.67%) <0.01 (<0.01–0.02)	19 (51.35%) < 0.01 (< 0.01–0.31)	22 (35.48%) < 0.01 (< 0.01–0.06)	37 (59.68%) 0.02 (< 0.01–0.22)
Minor_phyla	27 (75%) 0.03 (<0.01–0.45)	21 (56.76%) 0.01 (< 0.01–0.11)	41 (66.13%) 0.02 (< 0.01–0.17)	41 (66.13%) 0.03 (< 0.01–0.19)
Minor_genera	36 (100%) 2.05 (0.89–5.45)	37 (100%) 3.98 (1.31–7.91)	62 (100%) 1.86 (0.67–7.64)	62 (100%) 2.76 (1.12–7.92)
Unclassified_phyla*†	27 (75%) 0.09 (<0.01–0.45)	21 (56.76%) 0.01 (< 0.01–0.17)	55 (88.71%) 0.20 (0.03–0.66)	40 (64.52%) 0.03 (< 0.01–0.08)
Unclassified_genera	36 (100%) 0.58 (0.15–1.97)	37 (100%) 1.26 (0.38–3.19)	62 (100%) 1.03 (0.33–4.89)	61 (98.39%) 1.49 (0.25–4.83)

*n (%): number of samples where the phylum/genus was detected (relative frequency of detection), IQR, interquartile range.*

Wilcoxon rank sum test pairwise comparison with Bonferroni correction. Statistical differences were highlighted with: *within control group comparison, 1st vs. 5th week postpartum p < 0.05; *^†^within control group comparison, 1st vs. 5th week postpartum p < 0.001, **overall between group comparison: study vs. control group p < 0.01.

**FIGURE 3:**
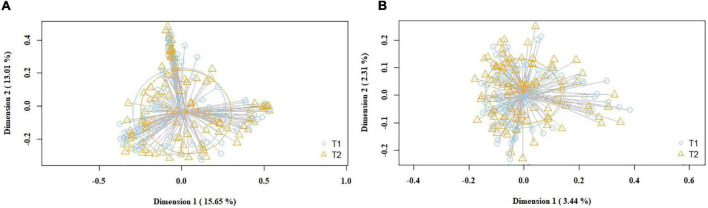
PCoA plots of bacterial profiles (at the ASVs level) of pooled (study and control group) milk samples according to time, based on the Bray-Curtis similarity (relative abundance) **(A)** and Jaccard’s coefficient for binary data (presence/absence) **(B)** analyses. Where blue circles (T1) refer to 1st week samples, and yellow triangles (T2), to 5th week samples. The value given on each axis label represents the percentage of the total variance explained by that axis.

Subsequently, the potential variation of the milk bacteriome composition over time was assessed. For this purpose, all the samples obtained at 1st week were compared with those collected at 5th week postpartum. This approach revealed that alpha diversity assessed by Simpson index increased [1st week 0.79 (0.67–0.85); 5th week 0.83 (0.74–0.87); *p* = 0.021]. Non-significant trends were observed in alpha diversity assessed by the Shannon index [1st week 2.19 (1.76–2.55); 5th week 2.3 (1.94–2.7); *p* = 0.210]. Beta diversity did not vary over time, neither using the Bray-Curtis distance matrix (*p* = 0.789) nor the binary Jaccard distance matrix (*p* = 0.934) (Figure 3). However, the analysis of specific bacterial taxonomic groups revealed a decrease in abundance of the phylum Firmicutes and the genus *Staphylococcus* over time and the opposite trends for several genera in the phylum Proteobacteria and other unclassified phyla (Table 3).

**TABLE 3:** Frequency of the most abundant bacterial phyla (shadowed boxes) and genera detected in the milk samples according to time.

	1st week postpartum		5th week postpartum		*P*-value^†^
		
Phylum/Genus	*n* (%)	Median (IQR)	*n* (%)	Median (IQR)	
Firmicutes	98 (100%)	90 (74.14–96.54)	99 (100%)	74.7 (23.01–95)	**<0.001**
*Streptococcus*	98 (100%)	36.27 (8.36–56.05)	99 (100%)	20.79 (1.85–51.52)	0.11
*Staphylococcus*	98 (100%)	34.63 (5.74–66.7)	99 (100%)	12.74 (2.64–32.96)	**0.001**
*Gemella*	59 (60.2%)	0.06 (<0.01–2.46)	74 (74.75%)	0.3 (<0.01–2.22)	0.26
*Bacillus*	26 (26.53%)	<0.01 (<0.01–0.01)	36 (36.36%)	<0.01 (<0.01–0.05)	0.092
*Lactobacillus*	60 (61.22%)	0.02 (<0.01–0.25)	67 (67.68%)	0.04 (<0.01–0.26)	0.62
*Enterococcus*	22 (22.45%)	<0.01 (<0.01–<0.01)	27 (27.27%)	<0.01 (<0.01–0.01)	0.49
*Lactococcus*	10 (10.2%)	<0.01 (<0.01–<0.01)	15 (15.15%)	<0.01 (<0.01–<0.01)	0.27
Proteobacteria	98 (100%)	4.53 (1.45–15.9)	99 (100%)	15.65 (2.25–67.75)	** <0.001**
*Acinetobacter*	85 (86.73%)	0.25 (0.07–1.2)	94 (94.95%)	0.9 (0.21–11.02)	** <0.001**
*Pseudomonas*	84 (85.71%)	0.3 (0.04–2.05)	85 (85.86%)	0.76 (0.17–6.25)	0.024
*Stenotrophomonas*	35 (35.71%)	<0.01 (<0.01—-0.03)	57 (57.58%)	0.03 (<0.01–0.61)	** <0.001**
*Serratia*	24 (24.49%)	<0.01 (<0.01–<0.01)	25 (25.25%)	<0.01 (<0.01–<0.01)	0.89
*Allorhizobium*	24 (24.49%)	<0.01 (<0.01–<0.01)	45 (45.45%)	<0.01 (<0.01–0.14)	**<0.001**
*Sphingomonas*	38 (38.78%)	<0.01 (<0.01–0.07)	46 (46.46%)	<0.01 (<0.01–0.06)	0.28
*Aquabacterium*	66 (67.35%)	0.05 (<0.01–0.27)	56 (56.57%)	0.01 (<0.01–0.08)	**0.009**
*Enterobacter*	9 (9.18%)	<0.01 (<0.01–<0.01)	21 (21.21%)	<0.01 (<0.01–<0.01)	**0.015**
*Sphingobium*	11 (11.22%)	<0.01 (<0.01–<0.01)	18 (18.18%)	<0.01 (<0.01–<0.01)	0.15
Actinobacteriota	92 (93.88%)	1.31 (0.3–3.76)	92 (92.93%)	0.63 (0.15–2.38)	0.062
*Rothia*	65 (66.33%)	0.27 (<0.01–2.09)	59 (59.6%)	0.03 (<0.01–0.44)	**0.038**
Bacteroidota	69 (70.41%)	0.07 (<0.01–0.45)	77 (77.78%)	0.1 (0.01–0.66)	0.21
*Chryseobacterium*	37 (37.76%)	<0.01 (<0.01–0.05)	56 (56.57%)	0.01 (<0.01–0.24)	**0.004**
Minor_phyla	68 (69.39%)	0.03 (<0.01–0.25)	62 (62.63%)	0.02 (<0.01–0.15)	0.34
Minor_genera	98 (100%)	1.94 (0.67–6.58)	99 (100%)	2.96 (1.19–7.95)	0.12
Unclassified_phyla	82 (83.67%)	0.13 (0.02–0.65)	61 (61.62%)	0.03 (<0.01–0.11)	** <0.001**
Unclassified_genera	98 (100%)	0.91 (0.26–2.78)	98 (98.99%)	1.36 (0.36–4.77)	0.18

*n (%): number of samples in which the phylum/genus was detected (relative frequency of detection). IQR, interquartile range.*

*^†^Wilcoxon rank tests with Bonferroni correction. The values in bold are statistically different.*

**FIGURE 4:**
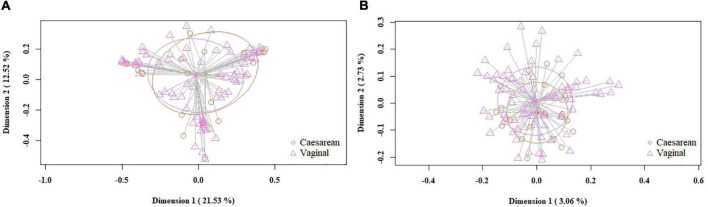
PCoA plots of bacterial profiles (at the ASVs level) of milk samples according to mode of labor completion, based on the Bray-Curtis similarity analysis (relative abundance) **(A)** and Jaccard’s coefficient for binary data (presence/absence) **(B)**. Where orange circles refer to samples from women who had cesarean section, and purple triangles, to those who had vaginal delivery. The value given on each axis label represents the percentage of the total variance explained by that axis.

When the samples were compared according to mode of labor completion (vaginal delivery vs. cesarean section), no statistical differences were found in relation to the biodiversity indices [Shannon and Simpson indices of 2.22 (1.78–2.53) and 0.78 (0.69–0.86), respectively, for the vaginal delivery group; Shannon and Simpson indices of 2.12 (1.6–2.56) and 0.80 (0.67–0.84), for the cesarean group]. Similarly, no statistical differences were observed concerning beta diversity neither using the Bray-Curtis distance matrix (*p* = 0.7413) nor the binary Jaccard distance (*p* = 0.526) (Figure 4). Analysis of the relative abundance of specific bacterial taxonomic groups showed a higher relative abundance of phylum Proteobacteria (*p* = 0.036) and genus *Pseudomonas*, *Stenotrophomonas* (*p* < 0.001) and *Serratia* (*p* = 0.002) among women who had cesarean section, and a higher abundance of the genus *Lactobacillus* among women with vaginal delivery (Table 4).

**TABLE 4:** Frequency of the most abundant bacterial phyla (shadowed boxes) and genera detected in the milk samples according to mode of delivery.

	Vaginal delivery		Cesarean section		*P*-value^†^
		
Phylum/genus	*n* (%)	Median (IQR)	*n* (%)	Median (IQR)	
Firmicutes	78 (100%)	90.8 (78.25–96.79)	20 (100%)	81.85 (51.76–93.62)	0.15
*Staphylococcus*	78 (100%)	34.63 (6.72–68.19)	20 (100%)	30.94 (3.93–52.69)	0.59
*Streptococcus*	78 (100%)	38.37 (9.62–56.05)	20 (100%)	11.12 (0.48–51.28)	0.1
*Gemella*	49 (62.82%)	0.15 (<0.01–2.83)	10 (50%)	0.01 (<0.01–1.08)	0.34
*Bacillus*	19 (24.36%)	<0.01 (<0.01–<0.01)	7 (35%)	<0.01 (<0.01–0.03)	0.34
*Anaerococcus*	12 (15.38%)	<0.01 (<0.01–<0.01)	2 (10%)	<0.01 (<0.01–<0.01)	0.62
*Enterococcus*	13 (16.67%)	<0.01 (<0.01–<0.01)	9 (45%)	<0.01 (<0.01–0.07)	**0.006**
*Lactobacillus*	51 (65.38%)	0.04 (<0.01–0.39)	9 (45%)	<0.01 (<0.01–0.05)	**0.045**
Proteobacteria	78 (100%)	3.79 (1.25–12.94)	20 (100%)	11.74 (3.91–36.34)	**0.036**
*Pseudomonas*	65 (83.33%)	0.24 (0.03–1.71)	19 (95%)	1.14 (0.29–8.48)	**0.007**
*Acinetobacter*	69 (88.46%)	0.24 (0.07–1.07)	16 (80%)	0.28 (0.07–1.54)	0.79
*Serratia*	14 (17.95%)	<0.01 (<0.01–<0.01)	10 (50%)	0.01 (<0.01–0.28)	**0.002**
*Stenotrophomonas*	21 (26.92%)	<0.01 (<0.01–0.01)	14 (70%)	0.04 (<0.01–0.94)	**<0.001**
*Haemophilus*	31 (39.74%)	<0.01 (<0.01–0.1)	5 (25%)	<0.01 (<0.01–0.01)	0.23
*Aquabacterium*	55 (70.51%)	0.06 (<0.01–0.27)	11 (55%)	0.02 (<0.01–0.27)	0.55
*Neisseria*	19 (24.36%)	<0.01 (<0.01–<0.01)	2 (10%)	<0.01 (<0.01–<0.01)	0.17
*Sphingomonas*	30 (38.46%)	<0.01 (<0.01–0.02)	8 (40%)	<0.01 (<0.01–0.24)	0.54
Actinobacteriota	75 (96.15%)	1.32 (0.37–4.31)	17 (85%)	0.74 (0.16–2.83)	0.2
*Rothia*	54 (69.23%)	0.3 (<0.01–1.94)	11 (55%)	0.01 (<0.01–2.2)	0.48
*Cutibacterium*	51 (65.38%)	0.04 (<0.01–0.09)	13 (65%)	0.04 (<0.01–0.15)	0.88
Bacteroidota	55 (70.51%)	0.08 (<0.01–0.45)	14 (70%)	0.04 (<0.01–0.32)	0.62
*Chryseobacterium*	23 (29.49%)	<0.01 (<0.01–0.01)	14 (70%)	0.01 (<0.01–0.06)	0.006
Minor_phyla	57 (73.08%)	0.03 (<0.01–0.24)	11 (55%)	0.01 (< 0.01–0.52)	0.46
Minor_genera	78 (100%)	1.43 (0.75–4.99)	20 (100%)	0.94 (0.16–2.94)	0.16
Unclassified_phyla	66 (84.62%)	0.13 (0.03–0.54)	16 (80%)	0.14 (0.01–1.4)	0.91
Unclassified_genera	78 (100%)	0.88 (0.26–2.45)	20 (100%)	1.08 (0.42–4.24)	0.58

*n (%): number of samples in which the phylum/genus was detected (relative frequency of detection). IQR, interquartile range.*

*^†^Wilcoxon rank tests with Bonferroni correction. The values in bold are statistically different.*

## Discussion

This study aimed to address potential differences on breast milk microbiota according to mother’s SARS-CoV-2 status, by means of the 16S RNA gene-based metataxonomic analysis. Overall, the results have shown great similarity in the structure of the bacterial populations in milk samples collected at first week and at fifth week of lactation, between the infected and the non-infected women. This suggests that peri-partum maternal SARS-CoV-2 infection does not have an impact on the human milk microbiota. On a recent report conducted on the same cohort of pregnant women (45), we have documented a distinct immunological profile in the human milk of SARS-CoV-2 infected mothers, consisting of higher concentrations of cytokines, chemokines and growth factors compared to that of negative women. Concentrations of most of the immune factors analyzed remained stable over time in SARS-CoV-2-positive women milk samples, but most of these compounds significantly decreased from the first to the fifth week postpartum in negative women. The results of both studies, together with the evidence of passive transfer of SARS-CoV-2-specific antibodies (4, 9, 10) and the lack of detection of this virus in most studies targeting human milk (2–4, 6, 45–48), reinforces the notion of safety and strengthens the current recommendation of breastfeeding in SARS-CoV-2-positive mothers.

*Staphylococcus* and *Streptococcus* were the most abundant bacterial genera and the only ones detected in all the milk samples. Such abundance of these two genera is in accord with previous studies (32, 35, 37, 49, 50), therefore supporting these two species as the hard core of the human milk bacteriome. Several studies have shown that coagulase-negative staphylococci are very abundant in colostrum or feces of breast-fed infants during the first days after birth (51), and that their levels tend to decrease in the following weeks (49, 52–54), usually coinciding with a rise in enterobacteria detection (54). Although few significant differences were also found at the phylum or genus level, this can be due to the relatively high degree of interindividual variability that characterizes the human milk bacteriome (50, 55–59).

Human milk is a relevant source of bacteria to the infant gut (60–62), that have a strong and perdurable impact on infant gut colonization (26). Therefore, the finding that the bacteriome structure evolution in the samples obtained during the first month postpartum in the SARS-CoV-2-positive women mirrors that of the negative ones is of utmost importance. So far, studies addressing the impact of maternal infections on the microbiological composition of milk are very scarce. It has been described that human papilloma virus (HPV) infection does not modify the bacterial composition of milk (63), although the authors declared that their results were not conclusive because of the low sample size (only 3 out of 35 samples analyzed were from HPV positive mothers). Breastfeeding has been repeatedly acknowledged as providing a certain degree of protection against mother-to-infant transmission of some viruses, including HIV (64). Genome analysis of a *Ligilactobacillus salivarius* strain with a high ability to inhibit *in vitro* HIV infectivity (65) revealed the existence of a mechanism by which such strain could interfere with gp120 attachment to immune cells (66). Interestingly, such strain had been isolated from human milk of a HIV-positive woman (with a high HIV titer in milk) whose breastfed infant remained uninfected during exclusive breastfeeding period.

The potential roles of the human milk microbiota in infants’ protection against life-viral infections (HIV, Zika, Ebola, cytomegalovirus…) or in minimizing their impact on health remains yet unexplored. It has been suggested, however, that the outcomes after suffering neonatal viral infections may be influenced by the interactions established between human milk oligosaccharides and the human milk and infant gut microbiomes (67).

Regardless of maternal SARS-CoV-2 status, our study has shown that the bacteriome composition changed from the 1st week to the 5th week postpartum milk, which confirms previous observations (68, 69). Different factors have been suggested as drivers of these differences, including an increased permeability of the mammary tight junctions during the first days after birth, or the impact of accumulating exposures to the infant microbiota over time. The decrease in the relative abundance of *Staphylococcus* from colostrum to mature milk samples has also been described before (70).

In contrast, only small differences in the bacteriome composition and structure were found when women were compared depending on the mode of labor completion (vaginal delivery vs. cesarean section). Previous studies have reported contradictory results when addressing the influence of mode of delivery on the composition of the human milk bacteriome, ranging from non-significant differences in the bacteriome profile (71) to significant differences at a variety of taxon levels (57, 72–75). In our study, cesarean section was associated to an increased presence of *Pseudomonas*, which is in accord with previous reports (76), but also to *Serratia* and *Stenotrophomonas*. All these bacterial genera are very relevant in the Neonatal Intensive Care Unit setting; therefore, this additional finding of our study also arouses interest from the perspective of increasing knowledge of the risk factors for perinatal infection.

This study faces some limitations. First, although the sample size was enough to reach statistical significance, it was relatively small, making difficult to extrapolate the results. Second, mothers with severe SARS-CoV-2 infection were not included. In addition, some factors known to modify the breast milk microbiota, such as overweight and obesity, could not be assessed since only one overweighed woman (study group) was included in this study. On the other hand, there was no comparison between the microbiome of the breast milk samples and the fecal microbiome of the respective infants (since the later samples were not available). In addition, the analysis of the milk microbiome was limited to two samples from each woman, with an interval of approximately 1 month. This decision was taken after confirmation of the lack of viral RNA in any of the milk samples collected over time (45). We considered that this observation period was adequate to elucidate an eventual distinct evolution of the bacterial structure related to mother’s infection status and/or to the time passed after birth. On the other hand, a strength of this work is the systematic approach to both SARS-CoV-2 documented infection and control women.

In summary, the results of this study indicate that the bacterial structure and composition of the human milk is, overall, well preserved among the SARS-CoV-2-positive women, providing additional support to foster breastfeeding in this population. The finding is relevant in terms of safety and efficacy of breastfeeding in this context, given the important role of colostrum and mature milk microbes as one of the first colonizers of the human gut, with potential impact on long-term health outcomes of infants.

## Data Availability Statement

The original contributions presented in the study are publicly available. This data can be found here: NCBI, BioProject, PRJNA795855.

## Ethics Statement

The studies involving human participants were reviewed and approved by Ethical Committee of Clinical Research of La Paz University Hospital. The patients/participants provided their written informed consent to participate in this study.

## Author Contributions

NG-T, IC, RA, and CA participated in sampling management and analysis, drafted the initial manuscript, reviewed, and approved the final version. LS-G participated in designing the study, patient’s enrollment, data gathering and analysis, drafted the initial manuscript, reviewed, and approved the final version. AP and JR conceptualized, designed the study, participated in data analyses, drafted the initial manuscript, reviewed, and approved the final version. FC conceptualized, designed the study, reviewed, and approved the final version of the manuscript. RG-S, ML-A, MM-P, DE-V, and EC-A participated in patient’s enrollment, data gathering, reviewed, and approved the final version of the manuscript. All authors contributed to the article and approved the submitted version.

## Conflict of Interest

The authors declare that the research was conducted in the absence of any commercial or financial relationships that could be construed as a potential conflict of interest.

## Publisher’s Note

All claims expressed in this article are solely those of the authors and do not necessarily represent those of their affiliated organizations, or those of the publisher, the editors and the reviewers. Any product that may be evaluated in this article, or claim that may be made by its manufacturer, is not guaranteed or endorsed by the publisher.

## Footnotes

1
https://www.R-project.org
